# Case report: Intraabdominal infection of *Mycobacterium syngnathidarum* in an immunocompetent patient confirmed by whole-genome sequencing

**DOI:** 10.3389/fmed.2023.1265594

**Published:** 2023-10-05

**Authors:** Hu Ge, Xiongwei Liang, Qiuran Lu, Aixiang He, Peiwen Zhong, Jun Liu, Yan Yu, Honglian Song

**Affiliations:** ^1^Changsha KingMed Center for Clinical Laboratory, Changsha, Hunan, China; ^2^Rucheng County People's Hospital, Rucheng, Hunan, China; ^3^Guangzhou KingCreate Biotechnology Company Limited, Guangzhou, Guangdong, China

**Keywords:** intraabdominal infection, *Mycobacterium syngnathidarum*, non-tuberculous mycobacteria, pathogen diagnosis, whole-genome sequencing

## Abstract

**Background:**

The taxonomic group of non-tuberculous mycobacteria (NTM) encompasses more than 190 species and subspecies, some of which can cause pulmonary and extrapulmonary diseases across various age groups in humans. However, different subspecies exhibit differential drug sensitivities, and traditional detection techniques struggle to accurately classify NTM. Therefore, clinicians need more effective detection methods to identify NTM subtypes, thus providing personalized medication for patients.

**Case presentation:**

We present the case of a 47-year-old female patient diagnosed with an intraabdominal infection caused by *Mycobacterium syngnathidarum.* Despite computed tomography of the chest suggesting potential tuberculosis, tuberculosis infection was ruled out due to negative TB-DNA results for ascites fluid and sputum and limited improvement of lung lesions after treatment. Additionally, acid-fast staining and Lowenstein–Jensen culture results revealed the presence of mycobacterium in ascites fluid. Subsequent whole-genome sequencing (WGS) confirmed the DNA sequences of *Mycobacterium syngnathidarum* in colonies isolated from the ascites fluid, which was further corroborated by polymerase chain reaction and Sanger sequencing. Ultimately, the patient achieved a complete recovery following the treatment regimen targeting *Mycobacterium syngnathidarum*, which involved clarithromycin, ethambutol hydrochloride, pyrazinamide, rifampicin, and isoniazid.

**Conclusion:**

This is the first reported case of *Mycobacterium syngnathidarum* infection in humans. *Mycobacterium syngnathidarum* was detected by WGS in this case, suggesting that WGS may serve as a high-resolution assay for the diagnosis of different subtypes of mycobacterium infection.

## Introduction

Non-tuberculous mycobacteria (NTM) are ubiquitous in the environment, present in water, soil, and aerosols, and can cause infections in humans and aquatic animals (1–4). NTM represent more than 190 species that can be classified as slow-growers including *M. avium Complex* (MAC), *M. kansasii*, and *M. gordonae*, and fast-growers, such as *M. abscessus complex*, *M. fortuitum*, and *M. chelonae*, according to whether colonies formed within 7 days (5–11). Despite worldwide recognition of the increasing prevalence and morbidity associated with NTM infection, assays to identify the species of NTM are still lacking (12, 13).

Whole-genome sequencing (WGS) can directly identify the sequences of pathogens and has been applied to the diagnosis of clinical infectious diseases (14, 15). In this study, we report a case of intraabdominal infection in a 47-year-old woman who was negative for TB-DNA in ascites fluid, while the yellow-green bacteria that were similar to *Mycobacterium tuberculosis* grew in Lowenstein–Jensen medium on the 3rd day. At the same time, a large number of DNA sequences of *Mycobacterium syngnathidarum* were identified by WGS. Following a 3 months treatment regimen consisting of ethambutol hydrochloride, pyrazinamide, rifampicin, isoniazid, and clarithromycin, the patient’s condition improved.

To date, only one case of *Mycobacterium syngnathidarum* cultured from a clinically ill fish of the family Syngnathidae has been reported in PubMed (16). As far as we know, the present case represents the first case of intraabdominal infection caused by *Mycobacterium syngnathidarum* in humans.

## Case description

A previously healthy 47-year-old female patient presented to the Rucheng County People’s Hospital’s urology department on September 26, 2022 with abdominal pain for 21 days and aggravation for 1 day. The patient had a medical history of thyroiditis and hypertension, denied any history of infectious diseases such as tuberculosis, and had no history of blood transfusion, surgery, or trauma.

**Figure 1:**
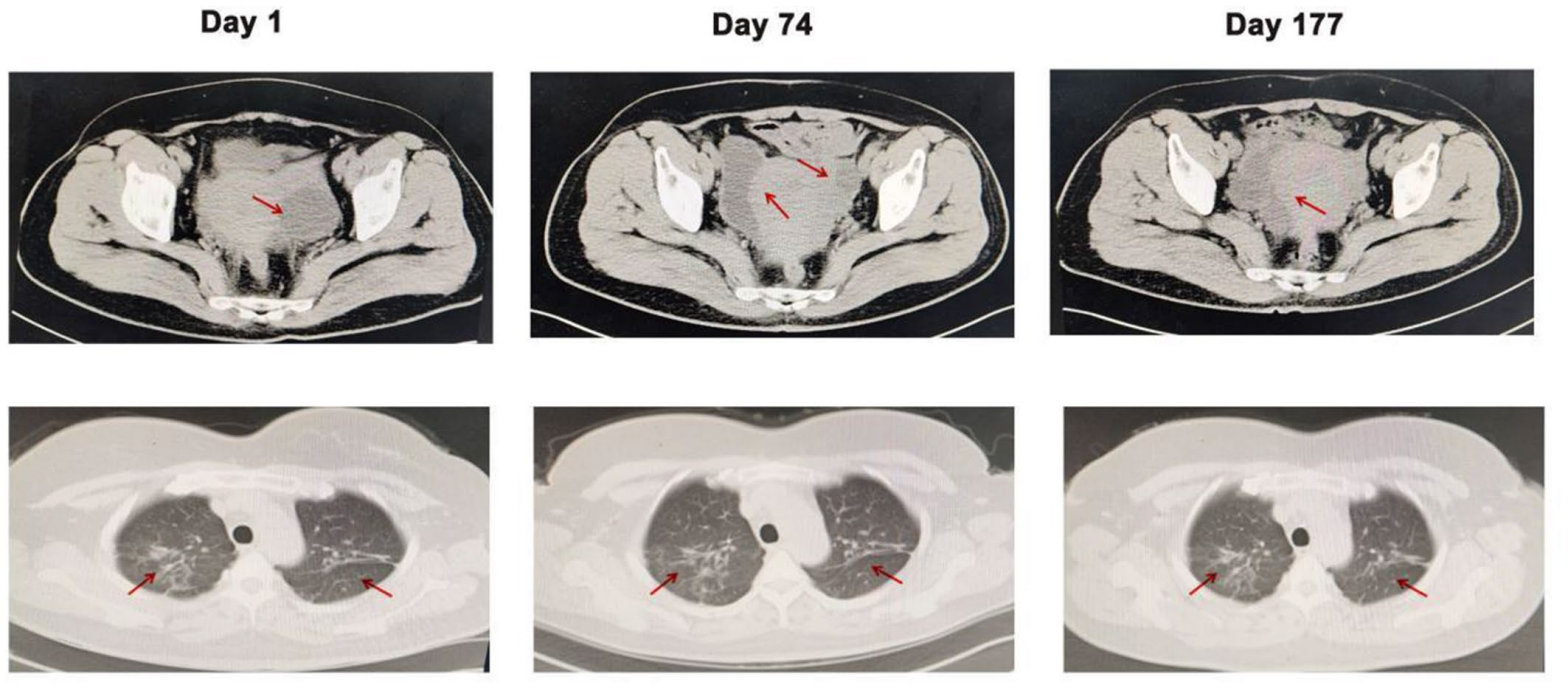
Computed tomography of the abdomen and chest on days 1, 74, and 177. Arrows indicate the lesions in the abdomen and lungs. Compared with the CT results on days 1 and 177, the low-density cystic lesion in the left adnexal area cavity disappeared, but patchy high-density lesions in both lungs did not improve.

On admission, the patient’s respiratory rate and body temperature were 20 /min and 36.5°C, respectively. Physical examination revealed a symmetrical and normal chest appearance with clear breath sounds in both lungs. However, the patient exhibited significant tenderness and rebound pain in the lower abdomen. The results of laboratory tests were as follows: The white blood cell (WBC) count was 11.65 × 10^9^/L, the percentages of neutrophils and lymphocytes were 89.6 and 5.10%, respectively, the C-reactive protein level was 34.03 mg/L, and the hemoglobin was 107.00 g/L. Procalcitonin concentration (PCT) and erythrocyte sedimentation rate (ESR) were normal. Color Doppler ultrasound showed a large amount of fluid in the abdominal cavity and a 57 × 52 mm cystic dark in the left adnexal area. At the same time, computed tomography (CT) of the chest and abdomen demonstrated patchy high-density lesions in both lungs. A large amount of effusion was observed in the abdominal cavity and pelvic cavity, along with a low-density cystic lesion of approximately 4.5 × 4.9 cm in the left adnexal area, along with an unclear and disarranged structure in the right adnexal area (Figure 1). These results suggested pulmonary tuberculosis and intraabdominal infection.

On the same day, the patient underwent laparoscopic exploration. Intraoperatively, it was found that the pelvic cavity contained approximately 400 mL of clear grass yellow fluid. The peritoneum, pelvic walls, and intestinal walls exhibited scattered grayish-yellow nodules. Adhesions were present between the intestinal duct, peritoneum, and the bilateral adnexa of the uterus. The left adnexa and the uterine basin wall formed an enveloped cyst with grass-yellow fluid inside. The surgeon aspirated fluid from the patient’s abdomen, separated the adhesions, aspirated fluid from the cyst, and excised the nodules in the greater omentum. Amoxicillin and clavulanate potassium tablets were given after the operation. Ascites fluid examination results were as follows: total protein, 55.81 g/L, glucose, 4.62 mmol/L, lactate dehydrogenase, 364.00 U/L, adenosine deaminase, 19 U/L, chlorine 100.3 mmol/L, and positive acid-fast staining. For further diagnosis of tuberculosis, ascites fluid was collected for Lowenstein–Jensen medium, acid-fast staining, and TB-DNA analysis, and sputum for Lowenstein–Jensen medium culture and acid-fast staining.

**Figure 2:**
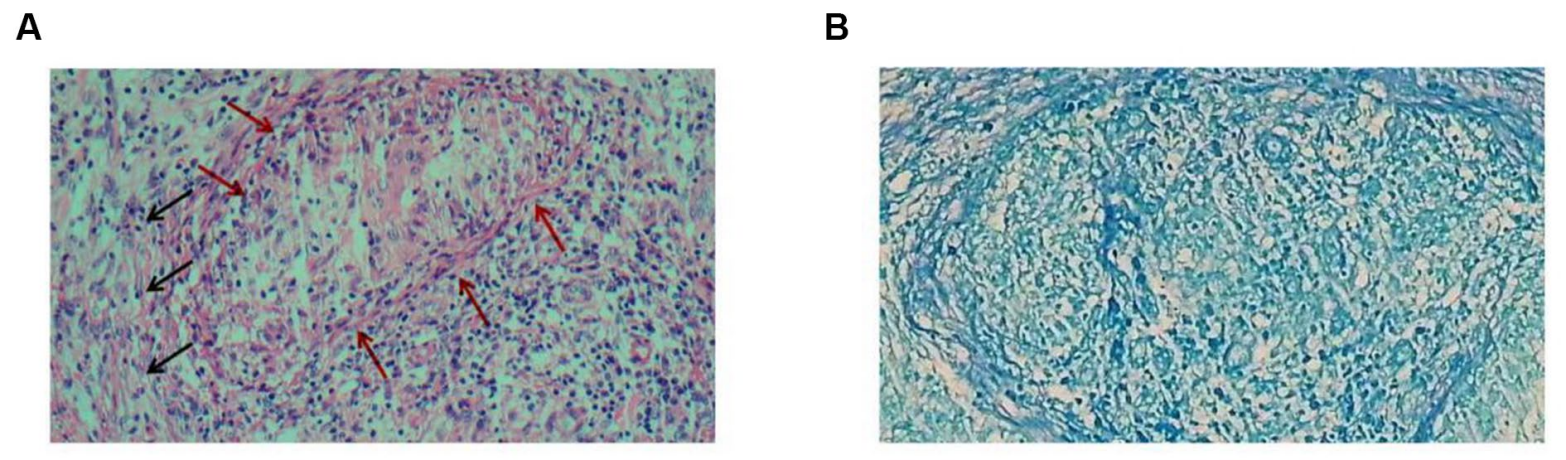
Histopathology of the nodular lesions on the greater omentum. **(A)** Hematoxylin–eosin staining shows hyperplasia of fibrous tissue (black arrows) with chronic granulomatous inflammation (red arrows). **(B)** Acid-fast staining was negative.

On the 2nd day, the histopathology of the greater omentum showed hyperplasia of fibrous tissue with chronic granulomatous inflammation, but acid-fast staining was negative (Figure 2). TB-DNA and acid-fast staining of the ascites fluid and sputum were negative. Based on the results from CT, pathology, and microscopic examination, the patient was diagnosed with tuberculous peritonitis and secondary tuberculosis, and anti-tuberculosis treatment was initiated with ethambutol hydrochloride, pyrazinamide, rifampicin, and isoniazid. On the 15th day, the patient’s condition improved and she was discharged from the hospital. Anti-tuberculosis treatment continued, and regular review was recommended.

**Figure 3:**
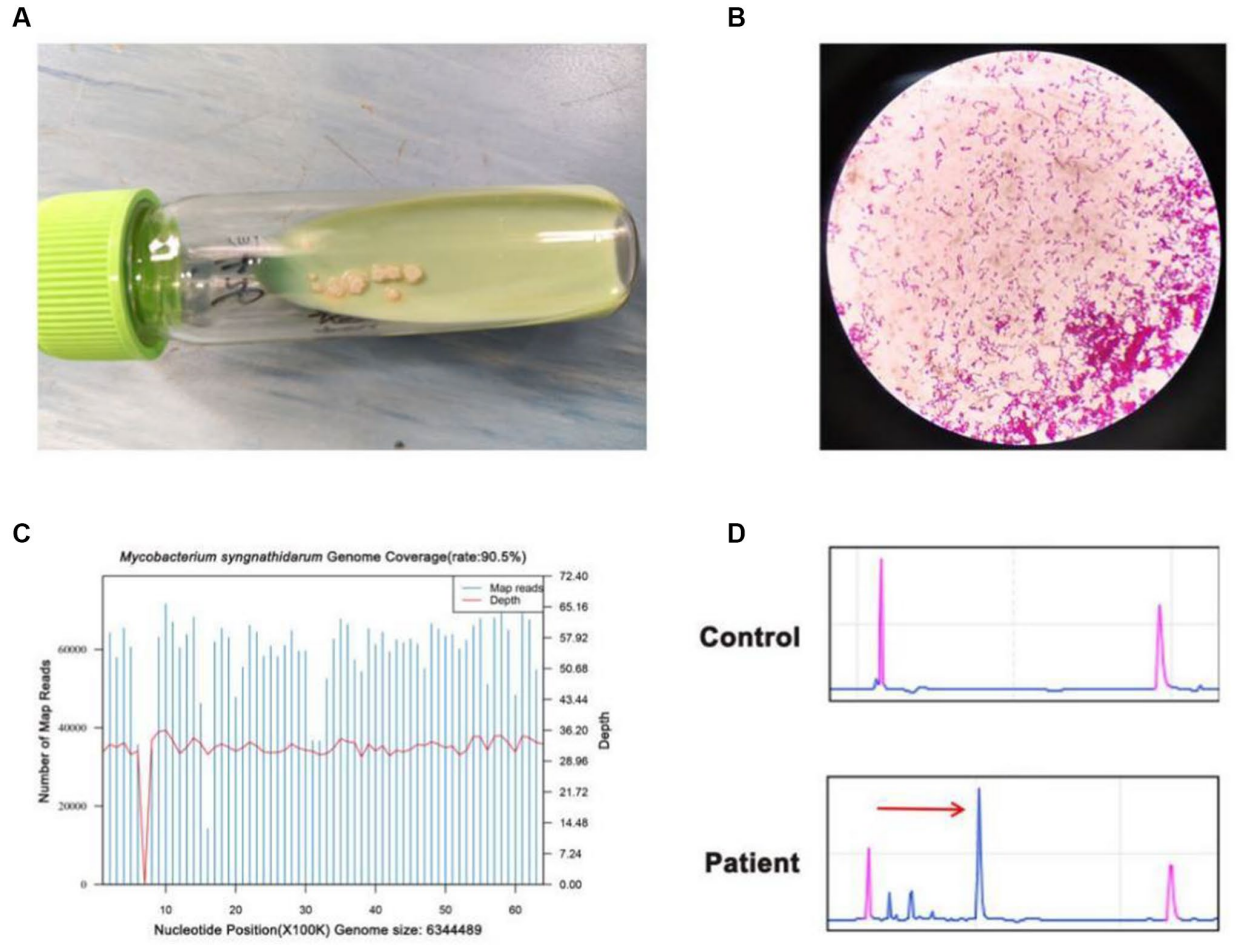
Identification of *Mycobacterium syngnathidarum* from the patient’s ascites fluid. **(A)** *Mycobacterium tuberculosis* grew in the ascites fluid of Lowenstein–Jensen medium on the 12th day. **(B)** The acid-fast staining of *Mycobacterium syngnathidarum*. **(C)** The WGS result showed that the coverage of *Mycobacterium syngnathidarum* was 90.5%. **(D)** Polymerase chain reaction and the capillary electrophoresis technique confirmed the *Mycobacterium syngnathidarum* infection in the patient.

On 14th October, the presence of yellow-green bacteria similar to *Mycobacterium tuberculosis* was observed in the ascites fluid culture after 12 days of incubation in the Lowenstein–Jensen medium (Figure 3A). The acid-fast staining was positive (Figure 3B), and single colonies were successfully grown after 3–4 days of bacteria isolation on blood agar plates and Lowenstein–Jensen medium. To gain further insight, WGS was performed on the cultures on 19th October, and the sequences of *Mycobacterium syngnathidarum* (ANI = 99.4282) were identified (Figure 3C). To validate the result of WGS, targeted PCR was performed on the bacteria isolated from the Lowenstein–Jensen medium using a pair of primers: forward 5′-ATGAGCGGTTCGGTGATGTT-3′, reverse 5′-CTACTCGCCAAATTCGCAGC-3′, targeting the genome of *Mycobacterium syngnathidarum*. The primers were designed and verified using Primer-BLAST^1^ based on the reference genome sequence of *Mycobacterium syngnathidarum* available in NCBI.^2^ The capillary electrophoresis technique (Qsep 100™; Bioptic) and Sanger sequencing confirmed the presence of *Mycobacterium syngnathidarum* (Figure 3D). However, due to the lack of reported pathogenicity with *Mycobacterium syngnathidarum* and the patient’s symptoms being alleviated after anti-tuberculosis treatment during hospitalization, no immediate adjustment to the treatment plan targeting *Mycobacterium syngnathidarum* was made.

On 8th December, the patient came to the hospital for reexamination as scheduled, prompting a review of CT and Color Doppler ultrasound. Compared to the CT result on 26 September, the abdominal effusion and pelvic effusion were observed. The cystic lesion in the left adnexal area shrank, but the lesion on the right side was enlarged (Figure 1). Combined with the patient’s symptoms and the previous results, the diagnosis of an intraabdominal infection caused by *Mycobacterium syngnathidarum* was made. Clarithromycin was added to the original treatment (ethambutol hydrochloride, pyrazinamide, rifampicin, and isoniazid) according to BacDive’s guidance.^3^ On March 21, 2023, the patient reported no abdominal discomfort and abdominal CT showed that the lesion in the left adnexal area disappeared (Figure 1). She continued eradication therapy for 6 months at home. In order to evaluate the sensitivity of *Mycobacterium syngnathidarum* to drugs, the drug susceptibility tests (Trek Diagnostic Systems Ltd., United Kingdom) of *Mycobacterium syngnathidarum* on August 29, 2023 showed that amikacin, ciprofloxacin, clarithromycin, doxycycline, linezolid, minocycline, and moxifloxacin were sensitive (Table 1).

**Table 1:** Drug susceptibility tests of *Mycobacterium syngnathidarum* by microtiter broth dilution method.

Metabolite	Sensitivity (+/−)	MIC value
Amikacin	+	≤1 μg/mL
Cefoxitin	/	64 μg/mL
Ciprofloxacin	+	0.25 μg/mL
Clarithromycin	+	1 μg/mL
Doxycycline	+	0.25 μg/mL
Imipenem	/	16 μg/mL
Linezolid	+	8 μg/mL
Minocycline	+	1 μg/mL
Moxifloxacin	+	≤0.25 μg/mL
Tobramycin	−	8 μg/mL
Trimethoprim+Sulfamethoxazole	−	>8/152 μg/mL

Referring to the M24-A2 document of the Clinical and Laboratory Standards Institute (CLSI), the results were classified as sensitive (+), intermediate (/), and resistant (−). MIC stands for minimum inhibitory concentration.

## Discussion and conclusion

NTM are ubiquitous and can survive in various environmental conditions, presenting challenges in their diagnosis and treatment (17, 18). However, clinical testing for drug susceptibility in NTM is lacking, and empiric therapy is not the best treatment according to the speed of NTM growth (19). Different strains of NTM have different susceptibilities to drugs, highlighting the importance of strain identification through molecular examination for devising appropriate treatment plans (19, 20). WGS aims to analyze the entire genome of a single bacterial colony, which provides better species identification than 16S RNA or other target genes (19, 21). In this study, we report the first case of intraabdominal infection caused by *Mycobacterium syngnathidarum* in humans.

Distinguishing between tuberculosis and non-tuberculosis infections remains a challenging aspect of clinical diagnosis. In this case, the patient presented with a 21 days history of lower abdominal pain, and the findings from abdomen computed tomography, acid-fast staining of ascites fluid, and histopathology of greater omental tubercles raised the possibility of tuberculosis infection. However, the identification of fast-growing *Mycobacterium syngnathidarum* through Lowenstein–Jensen medium, WGS, and targeted PCR, along with negative TB-DNA results in sputum and ascites fluid, indicated a potential intraabdominal infection caused by *Mycobacterium syngnathidarum*.

In this case, we first treated the patient with ethambutol hydrochloride, pyrazinamide, rifampicin, and isoniazid for anti-tuberculosis therapy. After 73 days, combined with the results of reexamination CT and Color Doppler ultrasound, *Mycobacterium syngnathidarum* was identified in ascites fluid by WGS and targeted PCR. Clarithromycin was added to the treatment for NTM. On March 21, 2023, the patient reported no abdominal discomfort and was advised to continue taking the medicine. It is worth noting that after 5 months of anti-TB treatment, the patient’s pulmonary nodules did not change significantly. In addition, the patient had no symptoms of a respiratory infection or other evidence of etiology. At the same time, this study conducted drug susceptibility tests on *Mycobacterium syngnathidarum*, which further proved that the bacteria was sensitive to clarithromycin (Table 1). Ultimately, we concluded that the patient did not have a lung infection, but an intraabdominal infection caused by *Mycobacterium syngnathidarum*.

In conclusion, our study reports a patient with an intraabdominal infection caused by *Mycobacterium syngnathidarum* that was diagnosed using WGS. The patient showed successful recovery following treatment with clarithromycin, ethambutol hydrochloride, pyrazinamide, rifampicin, and isoniazid. WGS may be a high-resolution and sensitive assay for the diagnosis and surveillance of NTM infection.

## Data availability statement

The original contributions presented in the study are included in the article/supplementary material, further inquiries can be directed to the corresponding authors.

## Ethics statement

The studies involving humans were approved by Rucheng County People’s Hospital ethics Association. The studies were conducted in accordance with the local legislation and institutional requirements. The participants provided their written informed consent to participate in this study. Written informed consent was obtained from the individual(s) for the publication of any potentially identifiable images or data included in this article.

## Author contributions

HG: Writing – original draft, Writing – review & editing, Conceptualization, Data curation, Formal analysis, Investigation, Methodology, Software. XWL: Methodology, Writing – original draft. QRL: Conceptualization, Investigation, Writing – original draft. AXH: Conceptualization, Investigation, Software, Writing – original draft. PWZ: Data curation, Methodology, Writing – original draft. JL: Formal analysis, Visualization, Writing – original draft. YY: Conceptualization, Investigation, Software, Writing – review & editing. HLS: Conceptualization, Data curation, Formal analysis, Investigation, Methodology, Writing – review & editing.

## Footnotes

^1^ https://www.ncbi.nlm.nih.gov/tools/primer-blast/

^2^ https://www.ncbi.nlm.nih.gov/

^3^ https://bacdive.dsmz.de/strain/158439
